# Spatiotemporal Evaluation of Water Quality and Hazardous Substances in Small Coastal Streams According to Watershed Characteristics

**DOI:** 10.3390/ijerph19020634

**Published:** 2022-01-06

**Authors:** Han-Saem Lee, Su-Jin Lim, Byung-Ran Lim, Hong-Seok Kim, Heung-Soo Lee, Tae-Ung Ahn, Hyun-Sang Shin

**Affiliations:** 1 Department of Environment Energy Engineering, Seoul National University of Science & Technology, Seoul 01811, Korea; hansun213@seoultech.ac.kr (H.-S.L.); d8066530314@daum.net (S.-J.L.); limbr@seoultech.ac.kr (B.-R.L.); 2 Korea Testing and Research Institute, Gwacheon 13810, Korea; hskim@ktr.or.kr; 3 Gyeonggido Environmental Preservation Association, Suwon 16229, Korea; hsleewater@epa.or.kr; 4 Environment Solution Partners, Gwangmyeong 14348, Korea; ahntu@naver.com

**Keywords:** land use, season, pollutant sources, water basin, hazardous material

## Abstract

In this study, spatial and temporal changes of eight water quality indicators and 30 types of hazardous substances including volatile organic compounds (VOCs), semi-volatile organic compounds (SVOCs), pesticides, and inorganic matters for the small coastal streams along the West Coast of South Korea were investigated. In coastal streams with clear seasonal changes in water quality, larger watershed areas led to greater contamination by particulate matter (i.e., suspended solids, r = 0.89), and smaller watershed areas led to greater contamination by organic matter (i.e., BOD, r = −0.78). The concentration of VOCs and pesticides was higher in agricultural areas, and those of SVOCs and metals were often higher in urban areas. According to the principal component analysis (PCA), during the wet season, the fluctuation in the water quality of coastal streams was higher in urban areas than in agricultural areas. Furthermore, coastal streams in residential areas exhibited higher levels of SVOCs, and those in industrial areas exhibited higher levels of metallic substances. Based on these results, the spatial and temporal trends of water quality and hazardous substances were obtained according to watershed characteristics, thereby clarifying the pollution characteristics of small-scale coastal streams and the major influencing factors.

## 1. Introduction

Water is the most important resource. It is an essential element not only for humans but for all living things [1,2]. Water is essential to ensure global food security, and safe water availability and supply has become the basis for most social functioning [1,2,3]. Humans use various types of water, such as surface water, groundwater, and lake water, as resources, and appropriately manage these resources to secure safe water [3,4,5,6]. Meanwhile, coastal streams usually take the form of small narrow rivers that flow directly into the ocean [7,8,9]. The west coastal stream of South Korea possesses a relatively compact watershed size compared to rivers with multi-level tributaries flowing from deep inland [7]. This small-scale coastal stream has low utility as a drinking water source because of its low flux. However, it has been used as a drainage channel for intensive runoff in densely populated urban areas [8,9,10] or as a water source in agricultural areas [11]. Similar to river tributaries, small coastal streams are at risk of pollution because of their geographical proximity to pollutant sources [9,10]. In addition, they are often neglected by authorities because they drain into the sea within a short period, unlike inland streams that directly impact drinking water sources [12,13,14]. The water quality of small coastal streams has important implications for the management of coastal ecosystems and can be used to clarify small-scale water systems. In this regard, Destouni et al. [5] inferred that even small hydrologically unmonitored near-coastal catchment areas can carry large nutrient and pollutant loads into the sea at a magnitude similar to or greater than that of monitored river loads.

The water quality of small coastal streams is affected by various factors, such as climate, topography, land use, and distribution of pollutants [4,10,15,16,17]. In addition, the diversity of pollutant sources in stream watersheds can be attributed to land-use change, which has a considerable impact on the water quality of coastal streams [7,18]. Discharge from sewage treatment facilities, road runoff, and combined sewer overflows are major pollutant sources of coastal streams in urban areas [7]. In contrast, untreated sewage (caused by the lack of appropriate disposal systems), livestock manure, fertilizers, and pesticides are the major sources of coastal stream pollution in agricultural areas [10,16]. The water quality of small coastal streams is significantly affected by the inflow of pollutants. Accordingly, the water quality and watershed scale of streams can easily change depending on rainfall intensity [19]. Therefore, for improved management of the water quality of small coastal streams, it is necessary to obtain basic information on the spatiotemporal variations in water quality according to pollutant sources.

Hazardous substances are released into water systems by various industrial, residential, and agricultural activities [20,21,22]. These hazardous substances include industrial chemicals, antibiotics, pesticides, domestic sewage, pharmaceuticals, persistent organic pollutants (POPs), and heavy metals. In the past decade, more hazardous substances have been detected in water systems [21,22]. Therefore, it has become problematic to secure the safety of water resources [20,23]. In addition to quantitatively analyze water quality indicators, such as biological oxygen demand (BOD) and chemical oxygen demand (COD), the inflow of specific hazardous substances, such as phenol and phthalates, should be qualitatively analyzed for improved management of small coastal streams. Furthermore, the changes in the inflow of hazardous substances in small coastal streams according to land use and seasons should be further investigated to clarify the types of hazardous substances discharged, according to the pollutant source.

In the present study, the spatial and temporal changes in basic water quality indicators and 30 types of specific hazardous substances were investigated in 16 small coastal streams in the West Sea of South Korea. The major objectives of this study were to (1) determine the spatial and temporal characteristics of water quality in small coastal streams, (2) identify the changes in type and distributions of hazardous substances according to land use and season, and (3) investigate the differences in spatiotemporal trends according to the characteristics of small coastal streams. The results are expected to provide basic information to efficiently manage small coastal streams and ensure water safety.

## 2. Materials and Methods

### 2.1. Sampling Sites and Collection

**Figure 1:**
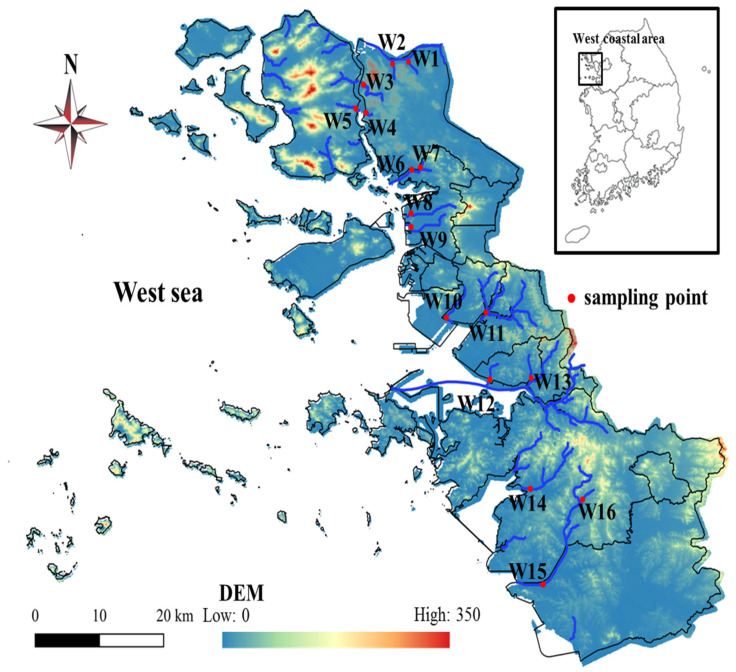
Digital elevation model (DEM) of the west coastal area and sampling site.

A total of 16 sampling sites (W1–W16) were selected in the West Sea coast of Gyeonggi-do (*N* 37°04′–71′ *E* 126°34′–88′), South Korea (Figure 1), where urban and agricultural lands are widely distributed. The selected coastal streams in this watershed have a maximum of two stages, and the watershed area of the stream ranges from 3.30 to 82.54 km^2^ (Table S1). The selected small coastal streams contain similar stream shapes, riverside vegetation, and soil, in addition to the presence of levee constructions along the riverside. In addition, the slopes of the coastal streams are low, so that pollutants flowing into the streams are discharged relatively slowly into the sea [24]. This coastal watershed is contaminated by a wide variety of pollutants, including household wastewater, treated wastewater from a sewage treatment plant, industrial wastewater, and unidentified non-point sources, such as roads, livestock, and agricultural runoff, which had remarkably compromised the water quality [24]. The water quality of the selected coastal streams was measured monthly from August 2019 to July 2020. All surface water samples were obtained during the mid-day time period of the day (i.e., 12:00–17:00) from the midstream unaffected by seawater and collected on a clear day in pre-washed translucent polyethylene terephthalate and 1 L amber brown glass bottles. The collected samples were immediately transferred to the laboratory and stored at 4 °C. The collection and preservation of samples followed the national method ES 04130.1c [25]. In addition, the analysis indicators were entirely measured within the preservation period (i.e., <1–7 days).

### 2.2. Water Quality and Hazardous Substances

Eight indicators were analyzed to evaluate the water quality of the coastal streams, namely pH, dissolved oxygen (DO), BOD, COD, total organic carbon (TOC), suspended solids (SS), total nitrogen (TN), and total phosphorus (TP). Each water quality indicator was measured in accordance with the South Korean guidelines for water pollution tests [25]. Detailed methods pertaining to analysis can be observed on the webpage of the Ministry of Environment [25]. The water quality grade of each indicator was based on the criteria of the Basic Law of Environmental Policy of South Korea [26], along with the SS and TN, which are not specified in the river criteria, but were based on lake criteria (Table S2). In addition, flow rates were also measured to evaluate seasonal differences.

A hazardous substance survey was conducted for four small coastal streams (W1, W4, W5, and W6) with high pollution levels and where the effects of pollutants could be easily distinguished through the field survey [24]. W4 and W6 are located in the urban area and presented the high-water pollution among the coastal streams studied. In addition, W1 and W5, are located in agricultural areas with an inflow of non-point sources. The investigated hazardous substances comprised the top 30 substances with the highest detection rate among 107 substances monitored in the Han River basin between 2007 and 2017 to determine the distribution of potentially toxic substances in the water system [27,28]. The selected hazardous substances were classified into four volatile organic compounds (VOCs: *o*-xylene, bromodichloromethane, bromoform, and toluene), seven semi-volatile organic compounds (SVOCs: phenol, fluorene, fluoranthene, dicamba, diethyl phthalate, dibutyl phthalate, and dinoseb), nine inorganic matters (perchlorate, Ba, Be, B, Mn, Mo, Se, Zn, and Ag), 10 pesticides (hexachlorobenzene, carbofuran, heptachlor epoxide A, heptachlor epoxide B, heptachlor, diuron, dieldrin, bisphenol-A, metolachlor, and quinoline). As displayed in Table 1, 16 organic hazardous substances were analyzed by gas chromatography–tandem mass spectrometry (GC-MS/MS, CP-3800/320-MS TQ, Varian, Agilent, Santa Clara, CA 95051, USA) based on the EPA 8270 method [29]. Perchlorates were analyzed using liquid chromatography–tandem mass spectrometry (LC-MS/MS, 6460 Triple Quad, Agilent, Santa Clara, CA 95051, USA) according to the standards for water pollution tests of South Korea [24]. The other organic hazardous substances were analyzed using LC-MS/MS (6460 Triple Quad, Agilent, USA) according to the EPA 549.2 method [30]. The metals were analyzed by inductively coupled plasma mass spectrometry (ICP-MS, CAP Q, Thermo, Waltham, MA 02451, USA) using the EPA 200.8 method [31]. The detailed operating conditions of the instrument are listed in Tables S3 and S4, and all analysis data used verified results according to standardized QA/QC test procedures related to the analysis of hazardous substances [29,30,31].

### 2.3. Statistical Analysis

All statistical analyses were conducted using the RStudio program (Boston, MA, USA), and all the variables were checked for normality (Shapiro–Wilk test, *p* > 0.05). Cluster analysis (CA) was conducted using the Ward’s method to investigate the differences in water quality according to the pollutant source [6]. In addition, *t*-test, ANOVA, and post-hoc (Scheffe’s method) tests were performed to identify differences between the clusters. Pearson’s analysis was performed for 26 indicators with secured normality to investigate the correlation between the variables (α < 0.05). We confirmed the suitability of PCA by Kaiser–Meyer–Olkin (KMO) and Bartlett’s test (KMO: *p* > 0.05, Bartlett’s: *p* < 0.05). Furthermore, PCA was conducted using over 1248 water quality data for 26 variables in small coastal streams. Variables that do not exhibit a normal distribution were excluded from the PCA analysis, and the variable data were used after normalization.

## 3. Results and Discussion

### 3.1. Water Quality by Season

**Figure 2:**
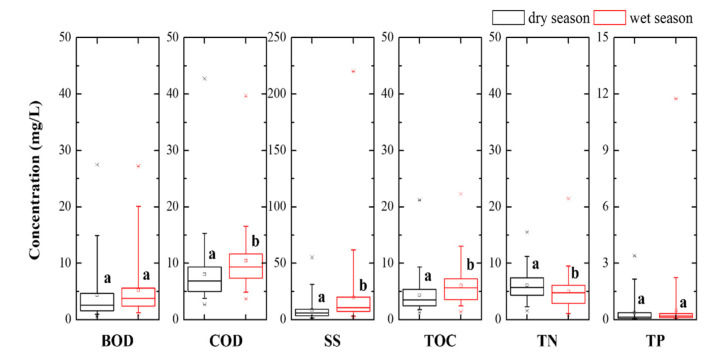
Boxplot of the concentration of organic (BOD, COD), particulate (TOC, SS), and nutrient (TN, TP) pollutants in coastal streams according to seasons. Lowercase letters (**a**,**b**) indicate significantly different groups (*p* < 0.05, *n* = 96).

The analysis of basic water quality indicators for the 16 coastal streams was divided into dry (October–March, average rainfall 46.5 mm/h) and wet seasons (April–September, average rainfall 155.3 mm/h) [32], as shown in Figure 2. Each water quality indicator was comparatively evaluated to identify the seasonal trends. BOD displayed an insignificant difference between the dry and wet season, but COD displayed higher average values in the wet season than in the dry season. The average values in the wet season (BOD 5.25 mg/L, COD 10.46 mg/L) were higher than the values for grade III (fair), which was set as the standard for coastal stream water quality in the West Coast [24]. For COD, the proportion of measured data exceeding grade III (i.e., >7 ppm) reached 81.3% in the wet season. This demonstrates that most small coastal streams were under significant organic contamination during the wet season.

The average TOC and SS values were higher in the wet than in the dry season, and both average values were higher than the limit of grade III in the wet season. The lowest proportion of SS exceeding grade III was observed at 10.4% in the dry season. It reached 30.2% in the wet season, which represents the highest increase rate among all indicators (Table S5). In addition, the coefficient of variation (CV) of the SS data was higher in the wet season, at 1.56, compared to other indicators (CV, 0.44–0.95), except for TP. Meanwhile, the TOC concentration was comparatively low even at high SS concentrations in the wet season. This indicates that the particulate matter inflow during the wet season possessed a relatively higher content of inorganic matter, such as soil and road sediment, compared to the dry season [33].

Similar to the above indicators, the average values of TN and TP also exceeded grade III limits in the wet season, and approximately half of them exceeded grade III values during the wet season. The average TN value was higher in the dry than in the wet season. The average TN in the dry season was 6.12 mg/L. In addition, 64.6% exceeded the grade III standard but decreased to 5.02 mg/L in the wet season. Considering that the decrease in TN during the wet season was low and its concentration of TN exhibited low variability (CV = 0.44) regardless of the season, its decrease in the wet season was attributed to a dilution effect caused by the flow rate increase. This change was greatest in W10, where the flow rate fluctuation was large (Table S5). Otherwise, Xu et al. [34] reported that TN concentrations might reach a fixed value (or range) in small shallow lakes receiving domestic sewage and farm drainage water by balancing the ecological water nutrients. This regulates algae and plant growth according to temperature, thereby mitigating eutrophication. The changes of TP were similar to those of SS, with a large variability (CV = 2.59) in the wet season, which appeared to be highly correlated with the inflow of particulate matter from non-point pollution sources during the wet season. Particularly, TP at W1 (agricultural area) was the highest, with an average of 2 mg/L (Table S5), and it was affected by pollutants, such as livestock manure and fertilizer utilization. In addition, among the coastal streams located in urban areas, W4 and W6 exhibited high values for all indicators, indicating a high degree of pollution.

### 3.2. Water Quality by Watershed Size

**Figure 3:**
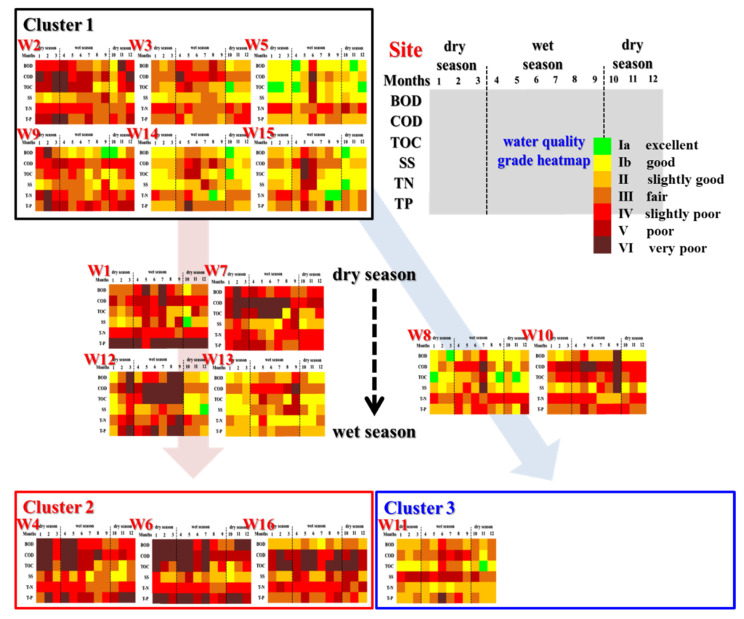
Heatmap showing the quality grade of coastal streams and seasonal changes in the cluster of coastal streams.

The CA was performed on the water quality data according to season, as displayed in Figure 3. The coastal streams were classified into three clusters. The ANOVA and post-hoc analysis (*p* < 0.05) confirmed that a significant difference exists between the water quality of the clusters. The water quality of Cluster 1 was significantly lower in all indicators, and was significantly higher in Cluster 2. Thus, Cluster 2 presented the highest pollution levels among all clusters (Table 2). The water quality of Cluster 3 was similar to that of Cluster 1, but its SS was significantly higher than that of Clusters 1 and 2. In addition, the average SS of Cluster 3 increased from 27.92 mg/L in the dry season to 41.83 mg/L in the wet season. Regardless of season, W2, W3, W5, W9, W14, and W15 belonged to Cluster 1; W4, W6, and W16 to Cluster 2; and W11 to Cluster 3. Some coastal streams shifted from Cluster 1 to Clusters 2 or 3 in the wet season. For instance, W1, W7, W12, and W13 shifted from Cluster 1 to 2, and W8 and W10 from Cluster 1 to 3. Clusters 2 and 3 presented high levels of contamination by organic and particulate pollutants, respectively, which suggests that organic and particulate matter represented their major pollutant inflow during the wet season. These differences in water quality were likely related to the watershed size rather than land use. The major land use pertaining to these areas with seasonal changes were urban and agricultural activities. The watershed area of coastal streams that changed from Cluster 1 to 2 was 3.30–14.97 km^2^, which is smaller than the watershed size of those that changed to Cluster 3 (18.77–33.58 km^2^). In addition, the average flow rates of the coastal streams that changed to Clusters 2 and 3 were 0.07 and 0.19 m^3^/s, respectively, which represents a significant difference in the flow rate (*t*-test, *p* < 0.05).

**Figure 4:**
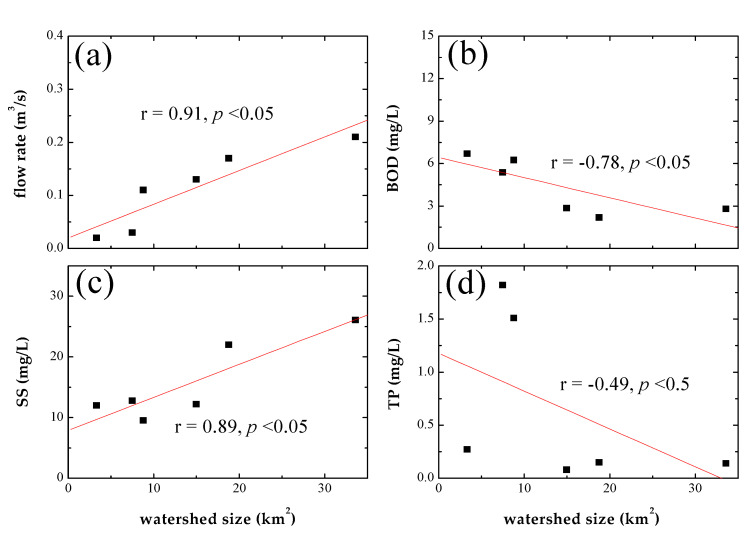
Relationship of the watershed size of the coastal streams, which show changes in cluster according to seasons (as shown in Figure 3, *n* = 6), with (**a**) flow rate, (**b**) BOD, (**c**) SS, and (**d**) TP.

To evaluate the correlation between seasonal water quality and watershed size during the wet season, Pearson correlation analysis was performed on six streams that changed clusters, using the mean value of the wet season, as shown in Figure 4. The flow rate (r = 0.91) and SS (r = 0.89) were significantly positively correlated with watershed size, whereas BOD, which represents organic pollutants, was significantly negatively correlated with it (r = −0.78). In contrast, TP was not significantly correlated to watershed size because of its large variability, but it demonstrated a negative correlation with it (r = −0.49). The increased flow rate in the stream seemed to contribute to the inflow of particulate matter, which is consistent with the results stating that SS was mostly composed of inorganic substances, such as soil and road sediment from non-point sources, in the wet season. Overall, the flow rate varied according to watershed size. Therefore, coastal streams with a relatively large flow rate (i.e., Cluster 3) were more likely to have a high inflow of particulate matter, such as soil and road sediments, from non-point sources. In contrast, coastal streams with a low watershed size (i.e., Cluster 2) were more likely to present an inflow of dissolved organic pollutants because of their low flow rate. These results indicate that the water pollution in these small coastal streams increased mainly because of the inflow of pollutants during the wet season. Accordingly, watershed size can be a major factor influencing the water quality of small coastal streams owing to its influence on non-point pollution sources during the wet season.

### 3.3. Changes in the Inflow of Hazardous Substances According to Season and Land Use

**Figure 5:**
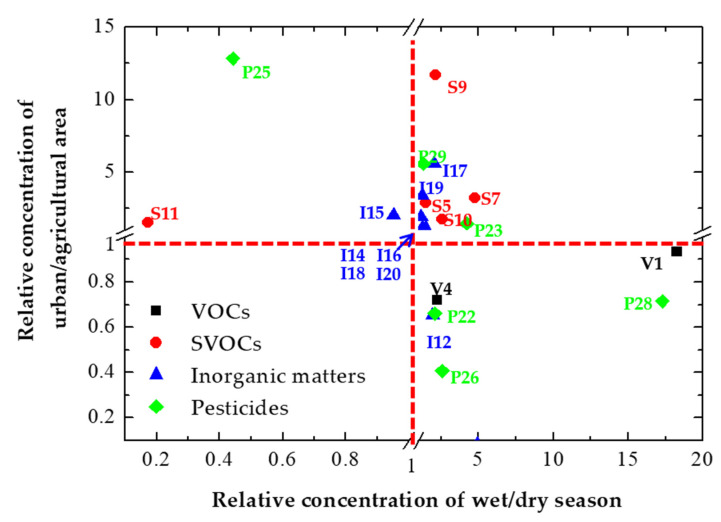
Distribution of relative concentrations of hazardous substances by season and land use (V; VOCs, S; SVOCs, I; Inorganic matters, P; Pesticides, the number after the alphabet indicates each hazardous substance, see Table 1).

To investigate the spatiotemporal characteristics of four small coastal streams according to land use, the seasonal distribution and potential sources of hazardous substances were investigated (Figure 5). Out of the total 30 investigated hazardous substances, S8 (dicamba), P21 (metolachlor), P27 (heptachlor), and P30 (quinoline) were not detected in the stream water samples. In addition, V3 (bromoform) and P24 (carbofuran) were detected only once throughout the study period (Table S6). The concentrations of all other hazardous substances were significantly lower than the internationally recommended criteria for drinking water [27,28]. Therefore, aquatic ecology and humans were at lower risk. However, different trends were observed depending on season and land use. The relative concentrations of V1 (*o*-xylene) and P28 (dieldrin) were significantly higher in the wet than in the dry season. In addition, the relative concentrations of S7 (fluoranthene), I12 (perchlorate), and P23 (hexachlorobenzene) were higher in the wet season as 4.2–4.9, suggesting that the hazardous substances are highly likely to be introduced from nonpoint sources. On the other hand, the relative concentrations of S11 (dinoseb) and P25 (heptachlor epoxide A) were < 1, with higher values in the dry than in the wet season. These results may indicate that the substances are continuously detected after exposure to water, considering that they are persistent organic pollutants (POPs) [27,28]. According to land use, in agricultural areas, no hazardous substances exhibited high concentrations in the dry season. However, six hazardous substances exhibited high concentrations in the wet season. These hazardous substances were related to pesticides (e.g., P22, P26, and P28), which are commonly used in agricultural areas. In addition, both V1 and P28 are known to be used as inert ingredients in agricultural crop products, post-harvest grain storage products, and residential pesticides [35]. In urban areas, the concentrations of SVOCs and inorganic matter were mostly high during the wet season, and SVOCs likely originated from non-point anthropogenic sources. The relative concentrations of S5 (phenol), S9 (diethylphthalate), and P25 were high, and higher concentrations were observed in urban areas. In this context, S5 is usually observed in complex wastewater from several industries, such as chemical, petrochemical, coke plant, and refineries [36]. In addition, S9 is widely utilized in plastics, coatings, and cosmetics [37,38]. Accordingly, S5, S9, and P25 displayed particularly high values in W4 (Table S7), where the industrial areas are distributed. In summary, the results confirmed that hazardous substances in coastal streams can be distributed differently depending on the season and land use, and most of the hazardous substances showed high concentrations in the urban watershed during the rainy season.

### 3.4. Spatiotemporal Trends by Pollutant Source

**Figure 6:**
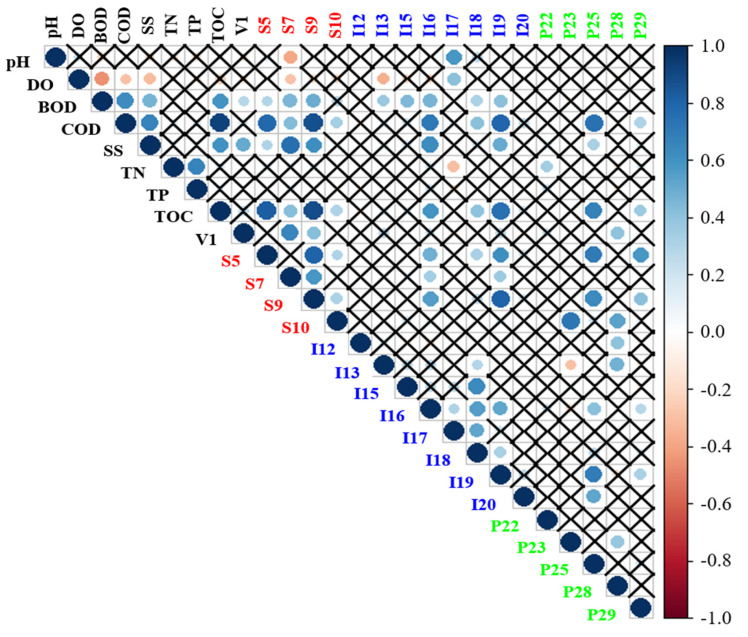
Pearson correlation between water quality indicators and hazardous substances. The size and color of the circles indicate the correlation coefficient values, and the cross indicates statistical insignificance (*p* > 0.05). (V; VOCs, S; SVOCs, I; Inorganic matters, P; Pesticides, the number after the alphabet indicates each hazardous substance, see Table 1).

To investigate the correlation between and among water quality indicators and hazardous substances, the Pearson correlation analysis was performed excluding the 12 hazardous substances with insufficient data (Figure 6). Among the water quality indicators, COD exhibited the highest correlation with TOC (r = 0.92, *p* < 0.05), and hazardous substances exhibited a significant correlation (r > 0.7, *p* < 0.05) with COD, SS, and TOC. COD was positively correlated with S5 (phenol), S9 (diethylphthalate), I16 (manganese), I19 (zinc), and P25 (heptachlor epoxide A); and TOC was positively correlated with S5, S9, and I19. Hazardous substances were highly correlated with indicators of recalcitrant organic matter and demonstrated a high concentration mostly in urban areas during the wet season (Figure 5). In addition, SS was positively correlated with S7 (fluoranthene), displaying a result consistent with previous studies confirming the fact that highly hydrophobic hazardous substances mostly exist in the adsorbed state on the particles rather than dissolved substances [39]. Among the hazardous substances, S5 displayed a significant positive correlation with S10 (r = 0.81, *p* < 0.05), and S9 exhibited a significant positive correlation with I19 (r = 0.80, *p* < 0.05), indicating that the pollutant sources of these hazardous substances could be similar and/or the fate of these three hazardous substances could be controlled by the same biogeochemical and physical processes [8,36].

**Figure 7:**
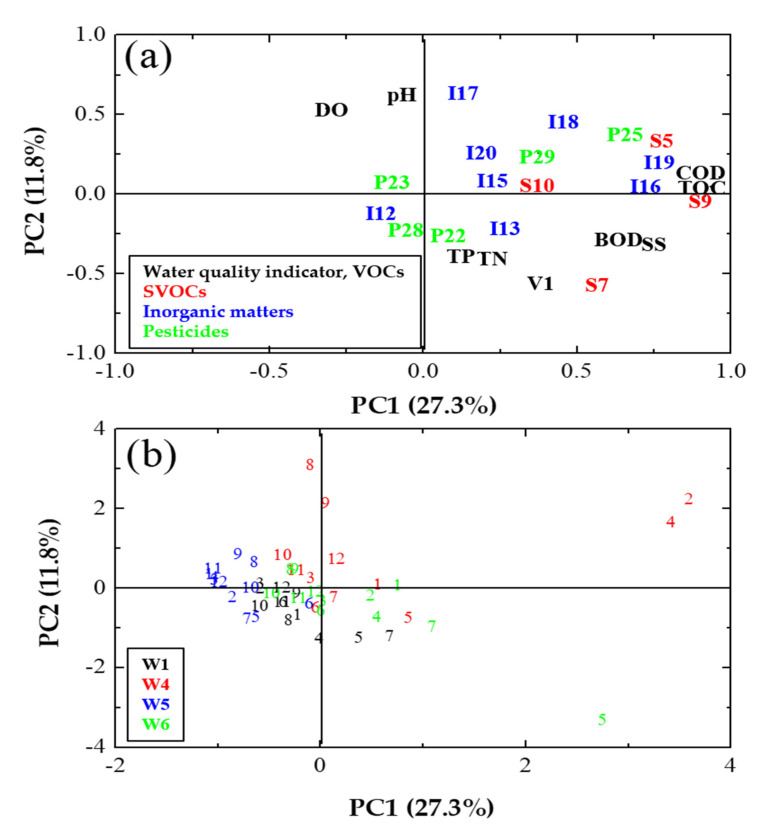
Distribution of (**a**) variables and (**b**) principal component analysis (PCA) results of the coastal streams with respect to the month. (**a**) V; VOCs, S; SVOCs, I; Inorganic matter, P; Pesticides, the number after the alphabet indicates each hazardous substance, see Table 1, (**b**) the number represents month (1 for January, 2 for February, and so on).

To further investigate the changes in water quality and inflow of hazardous substances according to pollutant source, PCA was conducted using water quality and hazardous substances as variables (Figure 7). Principal components 1 and 2 (PC1 and PC2) explained 27.3% and 11.8% of the total variations, respectively. Although the coverage of data variability was rather low to understand the entire data set, it was possible to confirm the water quality trend of coastal streams through each major component. PC1 displayed a positive relationship with most water quality indicators (i.e., COD, TOC, BOD, SS, TN, and TP) but a negative relationship with DO (Figure 7a). The loading of variables that cause oxygen depletion (e.g., COD, TOC) exhibited a positive value for PC1, whereas that for DO exhibit the opposite value, suggesting that a positive PC1 score represents the degree of water pollution. PC2 displayed a significant positive correlation with metal substances, such as I17 (molybdenum), I18 (selenium), and I20 (argentum), and a significant negative correlation with organic pollutants, such as V1 (*o*-xylene) and S7 (fluoranthene). Furthermore, S7, which is a PAH that can be discharged from roads and vehicles [36,37], was negatively correlated with organic water quality indicators. Therefore, PC2 was assumed to represent the type of hazardous substances. Specifically, we assumed that when PC2 was positive, the degree of contamination by inorganic matters was high, and when PC2 was negative, the influence of organic matter was high.

The comprehensive evaluation of monthly data confirmed that seasonal water quality fluctuations varied according to land use (Figure 7b). In W4 and W6, which are located in urban areas, the seasonal data fluctuation was larger than that for W1 and W5, which are located in agricultural areas. Coastal streams in urban areas displayed extremely high PC1 during the wet season compared to those in agricultural areas. As of January 2020, the water pollution level of the coastal streams increased but further decreased again during the wet season (Figure 7b). These results indicate that the water quality of coastal streams deteriorates because of the influx of pollutants during the wet season, but it is recovered in the dry season, further confirming that water quality is more deteriorated by the inflow of non-point pollutants from urban areas. As confirmed earlier, this is consistent with the result of high concentrations of specific hazardous substances (i.e., S5, S9, etc.) from urban areas during the wet season. However, in coastal streams of urban areas, the occurrence of hazardous substances seemed to differ depending on the main pollutant sources. For instance, in W4, which is near an industrial area [24], the inflow of metal substances was high during the wet season, whereas in W6, which is in a residential area [24], the high inflow of organic pollutants (i.e., S8, fluoranthene) likely originated from roads and agricultural land [40,41]. Therefore, the results confirmed that different pollution patterns and characteristics in this small coastal stream are undoubtedly affected by pollutant sources, such as industrial effluents, road runoff, and pesticides, rather than land use.

## 4. Conclusions

In this study, changes in the water quality of 16 small coastal streams and hazardous substances in four watershed areas were evaluated according to the characteristics of their respective watershed. We observed higher pollution levels in the wet than in the dry season. Particularly, coastal streams with a relatively large watershed area presented greater contamination by particulate matter (SS, r = 0.89, *p* < 0.05) during the wet season. In addition, greater pollution by organic matter (with BOD, r = −0.78, *p* < 0.05) was observed for smaller watersheds. Most hazardous substances also demonstrated higher concentrations in the wet season. Furthermore, the type of hazardous substances at higher concentrations changed depending on the pollutant source. VOC and pesticide levels, SVOC levels, and metal concentrations were higher in agricultural areas, residential areas, and industrial areas, respectively. These trends were more pronounced in the PCA results, which indicated a more evident inflow of pollutants during the wet season in urban areas regarding industrial and residential sources than in agricultural ones. Our results confirm that the spatiotemporal characteristics of water quality indicators and hazardous substances are highly influenced by watershed characteristics, such as climate, size, land use, and distribution of pollutants. Although this study has limitations regarding a wide variety of sampling points for monitoring of hazardous substances, the results also suggest that water system management in small coastal streams can be more easily performed by examining the characteristics of water pollution. In future research, it is expected that more various analyses can be conducted through water quality monitoring at multiple points exposed to more diverse pollutants.

## Figures and Tables

**Table 1:** Hazardous substance analysis indicators and analysis methods.

No.	Group	Compound	Analyzer	Pretreatment Method	Analysis Method	Analysis Group
V1	VOCs	*o*-xylene	GC/MS/MS	-	EPA 8270	A
V2	VOCs	bromodichloromethane	GC/MS/MS	-	EPA 8270	A
V3	VOCs	bromoform	GC/MS/MS	-	EPA 8270	A
V4	VOCs	toluene	GC/MS/MS	-	EPA 8270	A
S5	SVOCs	phenol	GC/MS/MS	EPA 5030C	EPA 8270	A
S6	SVOCs	fluorene	GC/MS/MS	EPA 5030C	EPA 8270	A
S7	SVOCs	fluoranthene	GC/MS/MS	EPA 5030C	EPA 8270	A
S8	SVOCs	dicamba	LC/MS/MS	-	EPA 549.2	C
S9	SVOCs	diethylphthalate	GC/MS/MS	-	EPA 8270	A
S10	SVOCs	di-*n*-butyl phthalate	GC/MS/MS	-	EPA 8270	A
S11	SVOCs	dinoseb	GC/MS/MS	-	EPA 8270	A
I12	Inorganic	perchlorate	LC/MS/MS	-	ES 04364.0	D
I13	Inorganic	barium	ICP/MS	EPA 5030C	EPA 200.8	B
I14	Inorganic	beryllium	ICP/MS	EPA 5030C	EPA 200.8	B
I15	Inorganic	boron	ICP/MS	EPA 5030C	EPA 200.8	B
I16	Inorganic	manganese	ICP/MS	EPA 5030C	EPA 200.8	B
I17	Inorganic	molybdenum	ICP/MS	EPA 5030C	EPA 200.8	B
I18	Inorganic	selenium	ICP/MS	EPA 5030C	EPA 200.8	B
I19	Inorganic	zinc	ICP/MS	EPA 5030C	EPA 200.8	B
I20	Inorganic	argentum	ICP/MS	EPA 5030C	EPA 200.8	B
P21	Pesticides	metolachlor	LC/MS/MS	-	EPA 549.2	C
P22	Pesticides	diuron	GC/MS/MS	EPA 5030C	EPA 8270	A
P23	Pesticides	hexachlorobenzene	GC/MS/MS	-	EPA 8270	A
P24	Pesticides	carbofuran	LC/MS/MS	-	EPA 549.2	C
P25	Pesticides	heptachlor epoxide A	GC/MS/MS	-	EPA 8270	A
P26	Pesticides	heptachlor epoxide B	GC/MS/MS	-	EPA 8270	A
P27	Pesticides	heptachlor	GC/MS/MS	-	EPA 8270	A
P28	Pesticides	dieldrin	GC/MS/MS	-	EPA 8270	A
P29	Pesticides	bisphenol A	LC/MS/MS	-	EPA 549.2	C
P30	Pesticides	quinoline	LC/MS/MS	-	EPA 549.2	C

**Table 2:** Mean value (mg/L) of water quality according to season and cluster.

Indicator	Dry Season			Wet Season		
	Cluster 1	Cluster 2	Cluster 3	Cluster 1	Cluster 2	Cluster 3
BOD	3.41 ^a^ *	9.05 ^b^	2.65 ^a^	3.36 ^a^	7.17 ^b^	4.54 ^a^
COD	6.93 ^a^	13.36 ^b^	5.30 ^a^	7.89 ^a^	13.12 ^b^	9.40 ^ab^
TOC	3.78 ^a^	7.68 ^b^	2.48 ^a^	4.67 ^a^	7.66 ^b^	5.05 ^ab^
SS	5.82 ^a^	12.95 ^b^	27.92 ^c^	11.54 ^a^	17.33 ^b^	41.83 ^c^
TN	6.23 ^a^	6.86 ^a^	2.59 ^b^	4.27 ^a^	6.03 ^a^	4.16 ^a^
TP	0.41 ^a^	0.37 ^a^	0.09 ^b^	0.26 ^a^	0.83 ^b^	0.21 ^a^

* Difference between lowercase letters within each group (quality indicator) between clusters means significant statistical difference (*p* < 0.05).

## Supplementary Materials

The following supporting information can be downloaded at: https://www.mdpi.com/article/10.3390/ijerph19020634/s1, Table S1: Sampling site and coastal stream stage; Table S2: Water quality grade depending on water quality indicators; Table S3: Instrument operating conditions de-pending on the analysis group (see the Table 2); Table S4: Instrument operating conditions of ICP-MS; Table S5: Mean and standard deviation of water quality grade of each indicator accord-ing to site and season; Table S6: Mean value (μg/L) of hazardous substances in coastal stream with respect to the month; Table S7: Mean value (μg/L) of hazardous substances with respect to the coastal stream site.

## Abbreviations

Volatile organic compounds (VOCs); semi-volatile organic compounds (SVOCs), principal component analysis (PCA); persistent organic pollutants (POPs); biological oxygen demand (BOD); chemical oxygen demand (COD); dissolved oxygen (DO); total organic carbon (TOC); suspended solids (SS); total nitrogen (TN); total phosphorus (TP); Gas chromatography–tandem mass spectrometry (GC-MS/MS); Liquid chromatography–tandem mass spectrometry (LC-MS/MS); Inductively coupled plasma mass spectrometry (ICP-MS); Cluster analysis (CA); Kaiser-Meyer-Olkin (KMO); coefficient of variation (CV).

## References

[1] Nasir J., Ashfaq M., Baig I.A., Punthakey J.F., Culas R., Ali A., Hassan F. (2021). Socioeconomic impact assessment of water resources conservation and management to protect groudwater in Punjab, Pakistan. Water.

[2] Ober J., Karwot J. (2021). Tap water quality: Seasonal user surveys in Poland. Energies.

[3] Nesheim I., Sundnes F., Enge C., Graversgaard M., van den Brink C., Farrow L., Glavan M., Hansen B., Leitão I.A., Rowbottom J. (2021). Multi-Actor Platforms in the Water–Agriculture Nexus: Synergies and Long-Term Meaningful Engagement. Water.

[4] Alnahit A.O., Mishra A.K., Khan A.A. (2020). Quantifying climate, streamflow, and watershed control on water quality across Southeastern US watersheds. Sci. Total Environ..

[5] Destouni G., Hannerz F., Prieto C., Jarsjö J., Shibuo Y. (2008). Small unmonitored near-coastal catchment area yielding large-mass loading to the sea. Glob. Biogeochem. Cycles.

[6] Liu L., Tang Z., Kong M., Chen X., Zhou C., Huang K., Wang Z. (2019). Tracing the potential pollution sources of the coastal water in Hong Kong with statistical models combining APCA-MLR. J. Environ. Manag..

[7] Lee H.-S., Shin H.-S. (2021). Evaluation of pollutants characteristics and effect of dissolved and particulate contaminants in tributaries of an urban watershed. Sci. Total Environ..

[8] Chen X., Zhou W., Pickett S.T.A., Li W., Han L. (2016). Spatial-temporal variations of water quality and its relationship to land use and land cover in Beijing, China. Int. J. Environ. Res. Public Health.

[9] Fan J., Jian X., Shang F., Zhang W., Zhang S., Fu H. (2021). Underestimated heavy metal pollution of the Minjiang River, SE China: Evidence from spatial and seasonal monitoring of suspended-load sediments. Sci. Total Environ..

[10] Lintern A., Webb J.A., Ryu D., Liu S., Waters D., Leahy P., Bende-Michl U., Western A.W. (2018). What are the key catchment characteristics affecting spatial differences in riverine water quality?. Water Resour. Res..

[11] Kourgialas N.N. (2021). A critical review of water resources in Greece: The key role of agricultural adaptation to climate-water effects. Sci. Total Environ..

[12] Myers D.T.L., Rediske R.R., McNair J.N., Parker A.D., Ogilvie E.W. (2021). Relating environmental variables with aquatic community structure in an agricultural/urban coldwater stream. Ecol. Process..

[13] Schliemann S.A., Grevstad N., Brazeau R.H. (2021). Water quality and spatio-temporal hot spots in an effluent-dominated urban river. Hydrol. Process..

[14] Xue D., De Baets B., Van Cleemput O., Hennessy C., Berglund M., Boeckx P. (2012). Use of a Bayesian isotope mixing model to estimate proportional contributions of multiple nitrate sources in surface water. Environ. Pollut..

[15] Gurjar S.K., Tare V. (2019). Spatial-temporal assessment of water quality and assimilative capacity of river Ramganga, a tributary of Ganga using multivariate analysis and QUEL2K. J. Clean. Prod..

[16] Mustapha A., Aris A.Z., Ramli M.F., Juahir H. (2012). Spatial-temporal variation of surface water quality in the downstream region of the Jakara River, north-western Nigeria: A statistical approach. J. Environ. Sci. Health A Tox. Hazard Subst. Environ. Eng..

[17] Vural A. (2020). Gündoğdu, A. High-Fluoride Risk and Toxicity in Surface Waters in Gümüşhane-Gökdere Valley Drainage Network (NE Turkey). J. Eng. Res. Appl. Sci..

[18] Larned S.T., Snelder T., Unwin M.J., McBride G.B. (2016). Water quality in New Zealand rivers: Current state and trends. N. Z. J. Mar. Freshw. Res..

[19] Wohl E. (2017). The significance of small streams. Front. Earth Sci..

[20] Chaturvedi P., Shukla P., Giri B.S., Chowdhary P., Chandra R., Gupta P., Pandey A. (2021). Prevalence and hazardous impact of pharmaceutical and personal care products and antibiotics in environment: A review on emerging contaminants. Environ. Res..

[21] Miglioranza K.S.B., Ondarza P.M., Costa P.G., de Azevedo A., Gonzalez M., Shimabukuro V.M., Grondona S.I., Mitton F.M., Barra R.O., Wania F. (2021). Spatial and temporal distribution of Persistent Organic Pollutants and current use pesticides in the atmosphere of Argentinean Patagonia. Chemosphere.

[22] Rathi B.S., Kumar P.S., Show P.L. (2021). A review on effective removal of emerging contaminants from aquatic systems: Current trends and scope for further research. J. Hazard. Mater..

[23] Wagner M., Andrew Lin K.Y., Oh W.D., Lisak G. (2021). Metal–organic frameworks for pesticidal persistent organic pollutants detection and adsorption—A mini review. J. Hazard. Mater..

[24] Ministry of the Environment (2013). Reports of Water Environment Management Plan for Han River West Sea. Han River Downstream Sihwa Lake Middle Watershed. https://www.me.go.kr/hg/web/board/read.do?pagerOffset=0&maxPageItems=10&maxIndexPages=10&searchKey=&searchValue=&menuId=3470&orgCd=&boardMasterId=136&boardCategoryId=&boardId=316339&decorator=.

[25] Ministry of the Environment (2017). Water Pollution Process Test Standards of South Korea.

[26] Ministry of Government Legislation South Korea (2019). The basic law of environmental policy South Korea. http://law.go.kr/.

[27] Hangang River Basin Management Committee. Report of Monitoring and Pathway Study of Hazardous Pollutants in the Tributaries of the Han River Basin..

[28] Hangang River Basin Management Committee. Report of Monitoring and Prediction System Development of Potentially Hazardous Organics in the Han River Basin..

[29] (2014). USEPA Method 8270E (SW-846).

[30] (1997). USEPA Method 549.2.

[31] (1994). USEPA Method 200.8.

[32] Korea Meteorological Administration (2021). Rainfall Statistics Survey. http://weather.go.kr/.

[33] Lee H.S., Hur J., Shin H.S. (2021). Dynamic exchange between particulate and dissolved matter following sequential resuspension of particles form an urban watershed under photo-irradiation. Environ. Pollut..

[34] Xu L., Li H., Liang X., Yao Y., Zhou L., Cui X. (2012). Water quality parameters response to temperature change in small shallow lakes. Phys. Chem. Earth Parts A B C.

[35] United States Environmental Protection Agency (USEPA) Inert Reassessment—Xylene; 2005. https://www.epa.gov.

[36] Wang Y., Chen H., Liu Y.X., Ren R.P., Lv Y.K. (2016). An adsorption-release-biodegradation system for simultaneous biodegradation of phenol and ammonium in phenol-rich wastewater. Bioresour. Technol..

[37] Huang M.Z., Ma Y.W., Wang Y., Wan J.Q., Zhang H.P. (2010). The fate of di-n-butyl phthalate in a laboratory-scale anaerobic/anoxic/oxic wastewater treatment process. Bioresour. Technol..

[38] Jin D., Kong X., Li Y., Bai Z., Zhuang G., Zhuang X., Deng Y. (2015). Biodegradation of di-*n*-butyl phthalate by *Achromobacter sp.* isolated from rural domestic wastewater. Int. J. Environ. Res. Public Health.

[39] Zhao N., Ju F., Pan H., Tang Z., Ling H. (2020). Molecular dynamics simulation of the interaction of water and humic acid in the adsorption of polycyclic aromatic hydrocarbons. Environ. Sci. Pollut. Res. Int..

[40] Shinya M., Tsuchinaga T., Kitano M., Yamada Y., Ishikawa M. (2000). Characterization of heavy metals and polycyclic aromatic hydrocarbons in urban highway runoff. Water Sci. Technol..

[41] Guzel B. (2021). Temporal variations and source identification of polycyclic aromatic hydrocarbons (PAHs) in rainwater collected in a semi-urban area within an industrial area in Turkey. Polycycl Aromat. Compd..

