# Supplementary material for: Spatiotemporal Evaluation of Water Quality and Hazardous Substances in Small Coastal Streams According to Watershed Characteristics
Source: Int J Environ Res Public Health. 2022 Jan 6;19(2):634. doi: 10.3390/ijerph19020634 (PMC8775941; doi:10.3390/ijerph19020634)
Supplement: Supplementary file 1 [file ijerph-19-00634-s001.zip › ijerph-1504001-supplementary.pdf]

Supplementary materials

Spatiotemporal Evaluation of Water Quality and Hazardous Substances in Small Coastal Streams According to Watershed Characteristics

Han-Saem Lee[1], Sy-Jin Lim[1], Byung-Ran Lim[1], Hong-Seok Kim[2], Heung-Soo Lee[3], Tae-Ung Ahn[4], and Hyun-Sang Shin[1,*]

[1] Department of Environment Energy Engineering, Seoul National University of Science & Technology; hansun213@seoultech.ac.kr, d8066530314@daum.net, limbr@seoultech.ac.kr
[2] Korea Testing and Research Institute; hskim@ktr.or.kr
[3] Gyeonggido Environmental Preservation Association; hsleewater@epa.or.kr
[4] Environment Solution Partners; ahntu@naver.com
* Correspondence: hyuns@seoultech.ac.kr; Tel.: +82-2-970-6625

Table S1. Sampling site and coastal stream stage.

no.		type	site	GPS		watershed area (km²)	coastal stream stage		
				longitude	latitude		main	primary	secondary
1	W1	surface water	Yangtaek	37°44'53" N	126°36'40" E	7.49	West sea	Yangtaek	-
2	W2	surface water	Gaehwa	37°44'42" N	126°34'56" E	9.76	West sea	Gaehwa	-
3	W3	surface water	Ponae	37°71'84" N	126°53'54" E	22.41	West sea	Ponae	-
4	W4	surface water	Seokjeong	37°68'57" N	126°54'17" E	4.39	West sea	Seokjeong	-
5	W5	surface water	Samdongam	37°70'09" N	126°46'96" E	32.50	West sea	Samdongam	-
6	W6	surface water	Geomdan	37°60'49" N	126°64'65" E	18.74	West sea	Geomdan	-
7	W7	surface water	Daepo	37°66'32" N	126°65'88" E	3.30	West sea	Geomdan	Daepo
8	W8	surface water	Gongchon	37°54'40" N	126°64'94" E	18.77	West sea	Gongchon	-
9	W9	surface water	Simgok	37°52'50" N	126°63'74" E	18.45	West sea	Simgok	-
10	W10	surface water	Seunggi	37°39'64" N	126°67'49" E	33.58	West sea	Seunggi	-
11	W11	surface water	Jangsu	37°43'27" N	126°73'56" E	19.64	West sea	Jangsu	-
12	W12	surface water	Singil	37°32'58" N	126°75'40" E	8.75	West sea	Singil	-
13	W13	surface water	Hwajeong	37°31'25" N	126°82'08" E	14.97	West sea	Ansan	Hwajeong
14	W14	surface water	Jaan	37°16'23" N	126°85'28" E	53.15	West sea	Jaan	-
15	W15	surface water	Balan	37°04'54" N	126°53'06" E	82.54	West sea	Balan	-
16	W16	surface water	Geumgok	37°11'62" N	126°88'99" E	9.43	West sea	Balan	Geumgok

Table S2. Water quality grade depending on water quality indicators.

grade	state	water quality indicator					
		BOD[a]	COD[a]	TOC[a]	SS[b]	TN[b]	TP[a]
Ia	excellent	≤ 1	≤ 2	≤ 2	≤ 1	≤ 1	≤ 0.02
Ib	good	≤ 2	≤ 4	≤ 3	≤ 5	≤ 2	≤ 0.04
II	slightly good	≤ 3	≤ 5	≤ 4	≤ 10	≤ 3	≤ 0.10
III	fair	≤ 5	≤ 7	≤ 5	≤ 15	≤ 5	≤ 0.20
IV	slightly poor	≤ 8	≤ 9	≤ 6	≤ 25	≤ 10	≤ 0.30
V	poor	≤ 10	≤ 11	≤ 8	≤ 100	≤ 15	≤ 0.50
VI	very poor	> 10	> 11	> 8	> 100	> 15	> 0.50

[a] from river criteria of the basic law of Envrionmental Policy, South Korea
[b] modified from lake criteria of the basic law of Envrionmental Policy, South Korea

Table S3. Instrument operating conditions depending on the analysis group (see the **Table 2**).

	group		
	A	B	D
instrument	GC-MS/MS	LC-MS/MS	LC-MS/MS
column	DB-5MS(Agilent, 30 m× 0.250 mm, 0.25um)	Poroshell 120 SB-C18, 2.7um 2.1 x 100mm	Eclipse plus c18 (3.0 × 150 mm, 3μm)
injector temp.	260 ºC	-	-
source temp.	280 ºC	-	-
column temp.		40 ºC	30 ºC
carrier gas/mobile phase	He	A : water (in 0.1% formic acid) B : acetonitrile	200mM methylamine solution in water
flow	2.25 mL/min	0.3 mL/min	0.35 mL/min

oven temp. program for analysis group A

Temp.(ºC)	Rate(ºC /min)	Hold time(min)
40	-	3
150	20	1
280	0	3

gradient condition for analysis group B

Time	A%	B%
0.0	95	5
0.5	85	15
1.0	60	40
8.0	2	98
10.0	2	98
10.1	95	5

Table S4. Instrument operating conditions of ICP-MS.

Instrument Parameters		Setting range for STD, KED sensitivity
Main	Torch Hoizontal	-1.3 mm
	Torch Vertical	-0.65 mm
	Nebulizer gas	1.1157 L/min
	Extraction Lens	-156.0 V
	CCT Focus lens	-0.24 V
Inlet	Peristaltic Pump Speed	40.0 rpm
	Spray Chamber Temperature	2.70 ℃
Plasma	Cool gas	14.0 L/min
	Auxilliary gas	0.8 L/min
	Plasma power	1550.0 W
Q-Cell	Collision gas 1	4.344 mL/min
	Collision gas 2	0.0 mL/min
	Pole Bias	-18.0 V
	CCT Bias	-21.0 V
Advanced	Extration Lens 1 Positive	0.0 V
	Focus Lens	-7.5 V
	Sampling Depth	5 mm
Gas	Ar	6 bar
	He	2 bar

Table S5. Mean and standard deviation of water quality grade of each indicator according to site and season.

site	Flowrate (m^3/s)		BOD (mg/L)		COD (mg/L)		TOC (mg/L)		SS (mg/L)		TN (mg/L)		TP (mg/L)	
	dry	wet	dry	wet	dry	wet	dry	wet	dry	wet	dry	wet	dry	wet
W1	0.017	0.025	3.78	5.38	8.10	9.38	4.44	4.97	5.31	12.8	7.79	7.74	2.21	1.82
	(0.008)[a]	(0.020)	(1.22)	(4.22)	(0.88)	(2.04)	(1.65)	(1.54)	(2.42)	(11.1)	(1.20)	(3.77)	(0.85)	(1.30)
W2	0.022	0.047	7.08	5.82	10.0	9.64	6.42	6.23	7.17	10.6	6.33	5.98	0.59	0.45
	(0.012)	(0.043)	(3.66)	(2.17)	(3.10)	(2.44)	(2.35)	(2.53)	(4.22)	(5.99)	(1.64)	(1.34)	(0.68)	(0.29)
W3	0.176	0.263	3.02	4.08	6.46	7.64	3.30	4.06	3.97	7.30	5.84	5.10	0.19	0.16
	(0.067)	(0.121)	(0.77)	(2.00)	(0.43)	(2.13)	(0.82)	(1.09)	(1.51)	(3.46)	(0.60)	(0.83)	(0.02)	(0.07)
W4	0.033	0.032	9.2	8.7	15.6	15.4	8.61	8.58	12.7	12.9	5.61	5.69	0.46	0.45
	(0.026)	(0.012)	(5.72)	(6.19)	(13.6)	(9.88)	(7.41)	(6.96)	(10.9)	(6.92)	(1.11)	(2.39)	(0.22)	(0.39)
W5	0.066	0.054	1.38	1.77	3.83	4.99	2.03	2.81	3.47	5.17	6.93	5.72	0.08	0.10
	(0.061)	(0.053)	(0.40)	(1.99)	(0.79)	(3.38)	(0.41)	(1.83)	(2.85)	(9.13)	(2.14)	(1.38)	(0.01)	(0.05)
W6	0.049	0.087	15.0	13.6	13.0	14.8	5.51	8.00	8.75	13.8	6.02	5.93	0.52	0.70
	(0.022)	(0.048)	(7.97)	(3.88)	(3.89)	(6.46)	(1.97)	(3.52)	(4.40)	(23.2)	(0.80)	(1.69)	(0.08)	(0.73)
W7	0.022	0.024	7.68	6.71	10.5	11.3	5.79	6.69	10.2	12.0	8.48	6.79	0.28	0.27
	(0.014)	(0.011)	(4.64)	(1.90)	(2.13)	(2.41)	(1.74)	(3.11)	(6.05)	(10.6)	(2.14)	(1.84)	(0.06)	(0.12)
W8	0.123	0.171	1.80	2.18	4.55	6.17	2.35	3.14	4.95	22.0	6.11	6.14	0.12	0.15
	(0.101)	(0.079)	(0.55)	(1.67)	(1.39)	(3.21)	(0.85)	(1.64)	(1.66)	(72.9)	(1.89)	(1.13)	(0.14)	(0.09)
W9	0.309	0.316	5.25	3.54	8.55	8.01	4.52	4.35	7.54	9.09	8.75	6.62	0.39	0.31
	(0.106)	(0.218)	(5.10)	(0.51)	(0.81)	(1.33)	(1.16)	(1.14)	(3.89)	(4.53)	(3.94)	(0.96)	(0.24)	(0.08)
W10	0.198	0.246	1.98	2.81	6.00	6.41	3.38	3.67	6.60	26.1	6.75	3.70	0.16	0.14
	(0.073)	(0.087)	(0.61)	(9.77)	(0.67)	(2.80)	(0.39)	(3.79)	(3.30)	(85.7)	(1.90)	(1.89)	(0.05)	(0.07)
W11	0.174	0.137	2.65	3.17	5.30	6.73	2.48	3.28	27.9	34.4	2.57	2.38	0.09	0.15
	(0.067)	(0.060)	(0.44)	(0.98)	(0.90)	(1.93)	(0.43)	(1.11)	(16.6)	(29.7)	(0.39)	(0.74)	(0.04)	(0.17)
W12	0.069	0.113	3.94	6.25	7.65	13.0	3.54	6.96	6.40	9.53	6.42	7.79	0.59	1.51
	(0.014)	(0.037)	(3.81)	(11.7)	(3.35)	(11.7)	(1.13)	(3.16)	(8.36)	(4.08)	(4.25)	(7.10)	(1.07)	(4.58)
W13	0.148	0.125	2.01	2.86	5.41	7.49	2.57	3.69	5.72	12.2	2.51	2.29	0.05	0.08
	(0.041)	(0.009)	(0.87)	(1.33)	(0.49)	(2.80)	(0.33)	(1.91)	(2.64)	(12.4)	(0.65)	(1.85)	(0.01)	(0.02)
W14	0.113	0.129	1.17	1.90	4.28	5.99	2.40	3.55	3.82	8.14	4.54	4.06	0.10	0.34
	(0.031)	(0.044)	(37)	(0.73)	(0.23)	(1.84)	(0.38)	(1.93)	(3.00)	(13.3)	(0.74)	(3.01)	(0.02)	(1.09)
W15	0.117	0.207	1.77	2.80	4.83	6.41	2.65	3.67	4.70	9.62	4.35	3.70	0.10	0.14
	(0.046)	(0.175)	(0.64)	(2.18)	(0.60)	(2.27)	(0.08)	(2.37)	(2.86)	(15.7)	(1.44)	(2.41)	(0.03)	(0.16)
W16	0.086	0.309	2.96	2.93	11.5	11.2	8.92	8.86	17.4	20.7	8.96	7.76	0.16	0.20
	(0.046)	(0.443)	(0.87)	(1.27)	(2.34)	(2.60)	(2.00)	(2.12)	(12.5)	(20.8)	(3.28)	(1.16)	(0.08)	(0.07)

[a] standard deviation

Table S6. Mean value (μg/L) of hazardous substances in coastal stream with respect to the month.

no.	compound	month (μg/L)											
		1	2	3	4	5	6	7	8	9	10	11	12
V1	*o*-xylene	ND[a]	0.0988	ND	0.2625	1.1210	0.0547	0.4619	0.0292	0.0927	0.0039	0.0064	0.0014
V2	bromodichloromethane	ND	ND	ND	ND	ND	0.0003	ND	ND	ND	ND	ND	ND
V3	bromoform	ND	ND	ND	ND	0.0004	ND	ND	ND	ND	ND	ND	ND
V4	toluene	0.0142	0.0122	ND	ND	0.0427	0.0094	0.0050	0.0017	0.0331	0.0089	0.0052	0.0005
S5	phenol	0.5914	1.2571	0.0853	2.7198	0.0751	0.0054	0.4948	0.0124	0.0334	0.1144	0.0050	0.2060
S6	fluorene	0.0041	0.0029	0.0017	0.0063	0.0059	0.0025	0.0070	ND	0.0100	0.0046	0.0036	0.0042
S7	fluoranthene	0.8659	0.5314	0.2300	1.3985	5.1284	2.5867	1.8042	0.0074	0.0123	0.0066	0.0036	0.6519
S8	dicamba	ND	ND	ND	ND	ND	ND	ND	ND	ND	ND	ND	ND
S9	diethylphthalate	0.1236	0.3507	0.0888	0.4417	0.6192	0.0894	0.2322	ND	0.0066	ND	ND	0.0785
S10	di-*n*-butyl phthalate	0.7359	1.4278	0.2490	0.9203	0.6192	0.5191	2.0384	3.3188	0.0432	ND	ND	0.4928
S11	dinoseb	0.0007	0.0007	0.0065	0.0004	0.0007	ND	ND	0.0004	ND	ND	ND	0.0003
I12	perchlorate	0.1192	0.4112	ND	ND	0.0558	ND	3.0856	ND	ND	ND	ND	0.1086
I13	barium	56.59	54.65	47.14	55.88	57.16	47.39	105.34	52.81	76.59	54.54	55.71	50.56
I14	beryllium	0.0063	0.0065	0.0053	0.0055	0.0178	0.0198	0.0110	0.0088	0.0118	0.0078	0.0070	0.0055

[a]not detected.

Table S6. Mean value (μg/L) of hazardous substances in coastal stream with respect to the month (continuous).

no.	compound	month (μg/L)											
		1	2	3	4	5	6	7	8	9	10	11	12
I15	boron	57.78	25.76	21.19	17.96	35.40	27.01	31.26	48.07	40.60	38.24	36.80	30.59
I16	manganese	175.99	292.04	226.18	313.04	306.44	178.70	312.06	220.37	192.18	209.58	202.91	186.77
I17	molybdenum	2.67	3.18	3.25	2.96	1.80	1.56	3.01	18.38	9.54	3.56	2.80	2.69
I18	selenium	1.27	0.81	0.55	0.78	0.50	0.46	1.01	0.82	1.28	0.78	0.79	0.61
I19	zinc	21.72	45.11	15.31	32.29	50.40	20.05	8.71	14.87	21.62	8.44	10.20	17.31
I20	argentum	0.0033	0.0223	0.0045	0.0030	0.0133	0.0098	0.0340	0.0143	0.0420	0.0250	0.0033	0.0203
P21	metolachlor	ND	ND	ND	ND	ND	ND	ND	ND	ND	ND	ND	ND
P22	diuron	0.0064	0.0153	0.0106	0.0526	0.0352	0.0100	0.0163	ND	ND	0.0083	0.0068	0.0075
P23	hexachlorobenzene	0.0003	0.0005	ND	0.0004	0.0038	0.0003	0.0006	ND	0.0024	0.0007	ND	0.0003
P24	carbofuran	ND[a]	ND	ND	ND	ND	ND	ND	0.2918	ND	ND	ND	ND
P25	heptachlor epoxide A	0.0224	0.0726	0.0120	0.0284	0.0110	0.0055	0.0076	ND	ND	ND	ND	0.0118
P26	heptachlor epoxide B	ND	ND	ND	ND	0.0005	0.0005	ND	ND	0.0008	0.0007	ND	ND
P27	heptachlor	ND	ND	ND	ND	ND	ND	ND	ND	ND	ND	ND	ND
P28	dieldrin	0.0107	ND	0.0005	0.0238	0.0389	0.0249	0.0896	0.0286	ND	0.0007	ND	ND
P29	bisphenol A	ND	ND	ND	0.0969	ND	0.0253	ND	ND	ND	0.0007	0.001	0.0905
P30	quinoline	ND	ND	ND	ND	ND	ND	ND	ND	ND	ND	ND	ND

[a]not detected.

Table S7. Mean value (μg/L) of hazardous substances with respect to the coastal stream site.

no.	compound	W1	W4	W5	W6
V1	*o*-xylene	0.1829	0.1906	0.3119	0.2722
V2	bromodichloromethane	ND[a]	0.0003	0.0003	0.0003
V3	bromoform	ND	ND	ND	0.0004
V4	toluene	0.0137	0.0075	0.0159	0.0138
S5	phenol	0.0257	1.1282	0.0196	0.3731
S6	fluorene	0.0034	0.0055	0.0016	0.0086
S7	fluoranthene	0.5991	0.9457	0.4494	2.4146
S8	dicamba	ND	ND	ND	ND
S9	diethylphthalate	0.0316	0.4813	0.0338	0.2853
S10	di-*n*-butyl phthalate	0.4508	1.1289	0.9685	1.3616
S11	dinoseb	0.0019	0.0029	0.0009	0.0013
I12	perchlorate	0.3231	0.0873	1.9713	0.1001
I13	barium	59.40	53.36	51.69	76.04
I14	beryllium	0.0110	0.0085	0.0116	0.0062
I15	boron	26.55	38.92	18.79	52.60
I16	manganese	260.02	342.70	61.38	274.64
I17	molybdenum	1.389	11.48	1.41	4.18
I18	selenium	0.64	1.42	0.2818	0.8637
I19	zinc	12.15	48.21	7.93	20.37
I20	argentum	0.0133	0.0203	0.0121	0.0092
P21	metolachlor	ND	ND	ND	ND
P22	diuron	0.0308	0.0155	0.0004	0.0051
P23	hexachlorobenzene	0.0014	0.0016	0.0008	0.0015
P24	carbofuran	ND	ND	0.2918	ND
P25	heptachlor epoxide A	0.0045	0.0626	0.0008	0.0065
P26	heptachlor epoxide B	0.0008	ND	0.0005	0.0005
P27	heptachlor	ND	ND	ND	ND
P28	dieldrin	0.0242	0.0269	0.0546	0.0294
P29	bisphenol A	0.0268	0.1917	0.0094	0.0112
P30	quinoline	ND	ND	ND	ND

[a]not detected.
